# Buccal Abscess With Dystrophic Calcification: A Case Report

**DOI:** 10.7759/cureus.81272

**Published:** 2025-03-27

**Authors:** Mai Kobayashi, Yasuyuki Fujii, Hinano Oda, Ayano Hatori, Daichi Chikazu

**Affiliations:** 1 Oral and Maxillofacial Surgery, Tokyo Medical University, Tokyo, JPN

**Keywords:** buccal abscess, dystrophic calcification, ectopic calcification, inflammation, ultrasonography

## Abstract

Dystrophic calcification, a type of ectopic calcification, refers to the pathological deposition of calcium ions in soft tissues. While cases have been reported in various regions of the body, its occurrence in the head and neck is rare. Here, we present a case of dystrophic calcification in the buccal mucosa. The patient, a 54-year-old man, initially visited his local dentist with complaints of swelling and pain in the right buccal mucosa, but the cause remained unidentified. He was subsequently referred to our department for further evaluation. At the initial visit, he exhibited swelling extending from the right buccal region to the mandible, along with redness and swelling in the right buccal mucosa near the premolars. A blood test revealed an elevated WBC count and increased CRP levels. USG showed hypoechoic areas in the right buccal region, raising suspicion of an abscess caused by infection or the presence of foreign material. The patient underwent an incision and drainage procedure, during which three tissue fragments were isolated. Pathological analysis confirmed that these were hard tissue structures distinct from teeth and bone, indicating dystrophic calcifications. Given that calcification can occur in the buccal region, it is essential to differentiate it from other conditions such as calcifying lymph nodes, phleboliths, sialolithiasis, and myositis ossificans. Accurate diagnosis requires a combination of radiological imaging, USG, clinical examination, and thorough medical history assessment.

## Introduction

Ectopic calcification refers to the abnormal deposition of minerals in soft tissues [1]. It is classified into five types: metastatic, tumoral, calciphylaxis, idiopathic, and dystrophic. Metastatic, tumoral, and calciphylaxis calcifications result from systemic mineral imbalances, leading to calcium deposition in previously normal tissues. Specifically, metastatic calcification is driven by elevated serum calcium levels due to malignancies or calcium metabolism disorders such as hyperparathyroidism. Tumoral calcification is a familial disorder commonly observed in patients with renal failure, while calciphylaxis is characterized by small vessel vasculitis and skin necrosis.

In contrast, idiopathic and dystrophic calcifications occur under normal calcium metabolism. The key distinction between the two lies in the presence of systemic electrolyte abnormalities in idiopathic calcification, whereas dystrophic calcification occurs in their absence [2]. Dystrophic calcification typically arises in response to trauma, infection, or inflammation, progressing without detectable abnormalities in blood calcium levels [3]. It follows a pathophysiological process similar to bone tissue mineralization [4]. Bone formation occurs in two stages: first, osteoblasts secrete extracellular matrix components, and second, mineralization occurs through the deposition of crystalline hydroxyapatite [5]. While these processes are generally restricted to hard tissues such as bones, teeth, and cartilage, regulatory mechanisms prevent calcium deposition in soft tissues. Disruptions in these systems, however, can lead to dystrophic calcification [6].

Previously, dystrophic calcification was considered a passive degenerative process caused by the precipitation of supersaturated calcium and phosphorus ions in degenerated cells due to aging, diabetes, atherosclerosis, or other conditions. However, emerging evidence suggests that it results from the abnormal differentiation of endothelial cells, smooth muscle cells, and fibroblast-like cells into osteoblast-like cells [7]. Whether these cells directly differentiate into osteoblasts or first revert to mesenchymal stromal cells before becoming osteoblasts remains unclear [8].

Dystrophic calcification most commonly affects the heart, skeletal muscles, and kidneys, typically following necrosis, inflammation, or infection [9]. In contrast, its occurrence in the head and neck region is rare [2]. When present, dystrophic calcifications in these areas are often asymptomatic and are usually discovered incidentally [10]. Here, we report a case of a man diagnosed with a buccal abscess associated with dystrophic calcification in the buccal mucosa.

## Case presentation

A 54-year-old man presented to our department with a chief complaint of pain and swelling in the right buccal region. He first noticed the swelling at the end of October and visited a nearby dentist, but the cause could not be identified, so he was referred to our department. This was the first time he had experienced such symptoms.

His medical history included disc herniation, narrowing of the disk space, and high blood pressure (174/120). He was not taking any medications and had no known allergies. He smoked 10 cigarettes per day and occasionally consumed alcohol.

Blood tests revealed elevated inflammatory markers, with a WBC count of 18.0 × 10³/µL and a CRP level of 1.62 mg/dL (Table 1).

**Table 1:** Summary of blood test results ALT: alanine aminotransferase; BUN: blood urea nitrogen; eGFR: estimated glomerular filtration rate; NEUT: neutrophil; ST: serum transaminase

Parameter	Result	Reference range
WBC	18.0 × 10³/µL	3.3-8.6 × 10³/µL
RBC	5.11 × 10⁶/µL	4.35-5.55 × 10⁶/µL
Hemoglobin	16.0 g/dL	13.7-16.8 g/dL
NEUT	84.30%	42.0-74.0%
ST	18 U/L	13-30 U/L
ALT	23 U/L	10-42 U/L
BUN	12.9 mg/dL	8.0-20.0 mg/dL
CRE	0.90 mg/dL	0.65-1.07 mg/dL
eGFR	69.3	-
CRP	1.62 mg/dL	0.00-0.14 mg/dL
Na	141 mEq/L	138-145 mmol/L
Cl	107 mEq/L	101-108 mmol/L
K	4.1 mEq/L	3.6-4.8 mmol/L
Ca	9.3 mg/dL	8.8-10.1 mg/dL

**Figure 1:**
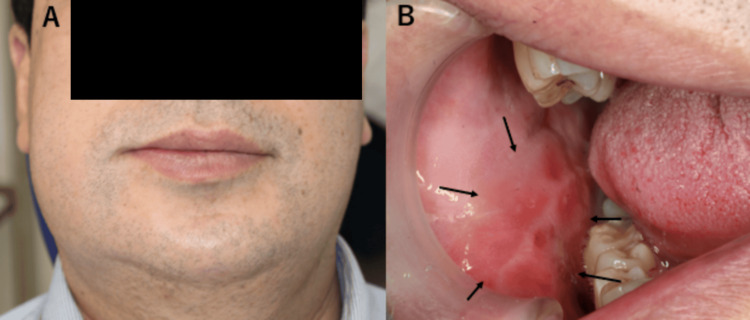
Physical findings of the patient (A) Facial appearance showing swelling extending from the right buccal region to the right mandible. (B) Intraoral photograph revealing internal bleeding and swelling of the buccal mucosa near the lower premolars.

Physical examination revealed swelling extending from the right buccal region to the right mandible (Figure 1A), along with internal bleeding and swelling of the buccal mucosa near the lower premolars. The swelling was located near the occlusal plane and showed an impression of the teeth (Figure 1B). No trismus or difficulty swallowing was observed.

Due to the patient’s obesity, palpation did not confirm the presence of an obvious abscess. However, given the strong clinical suspicion of cellulitis, several imaging and laboratory tests were performed.

**Figure 2:**
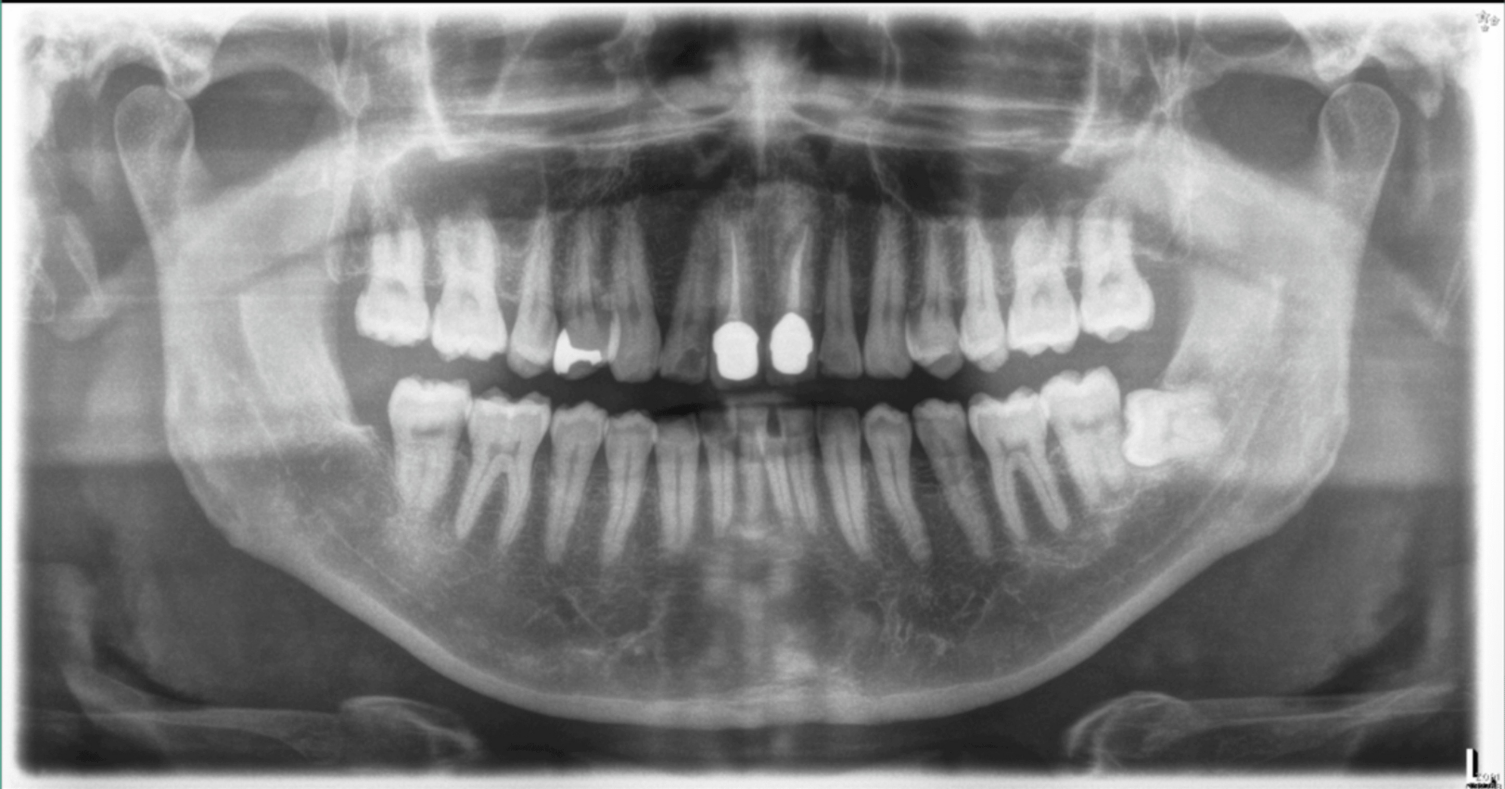
OPG showing no apparent abnormalities OPG: orthopantomography

Orthopantomography (OPG) revealed no apparent abnormalities (Figure 2).

**Figure 3:**
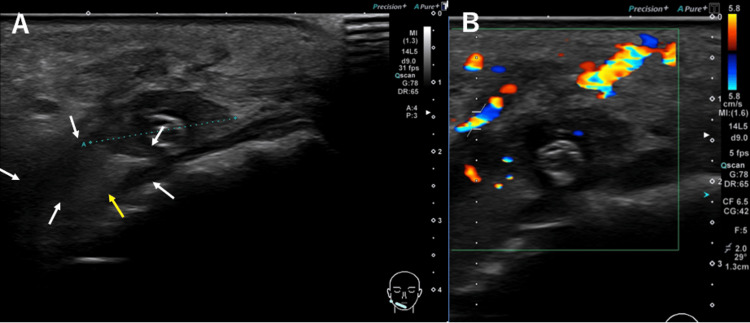
USG findings of the patient (A) Hypoechoic area (white arrows) measuring approximately 21 × 10 mm, with a central hyperechoic area (yellow arrow). (B) Blood flow signals detected around the hypoechoic area.

Contrast-enhanced CT (CECT) could not be performed due to the patient’s high blood pressure. USG revealed a hypoechoic area measuring approximately 21 × 10 mm, with a central hyperechoic region (Figure 3A) and a detectable blood flow signal (Figure 3B). Additionally, USG confirmed the presence of an abscess and foreign substances. Based on laboratory and clinical findings, a diagnosis of a right buccal abscess caused by infection was made.gnosis of right buccal abscess caused by the infection.

**Figure 4:**
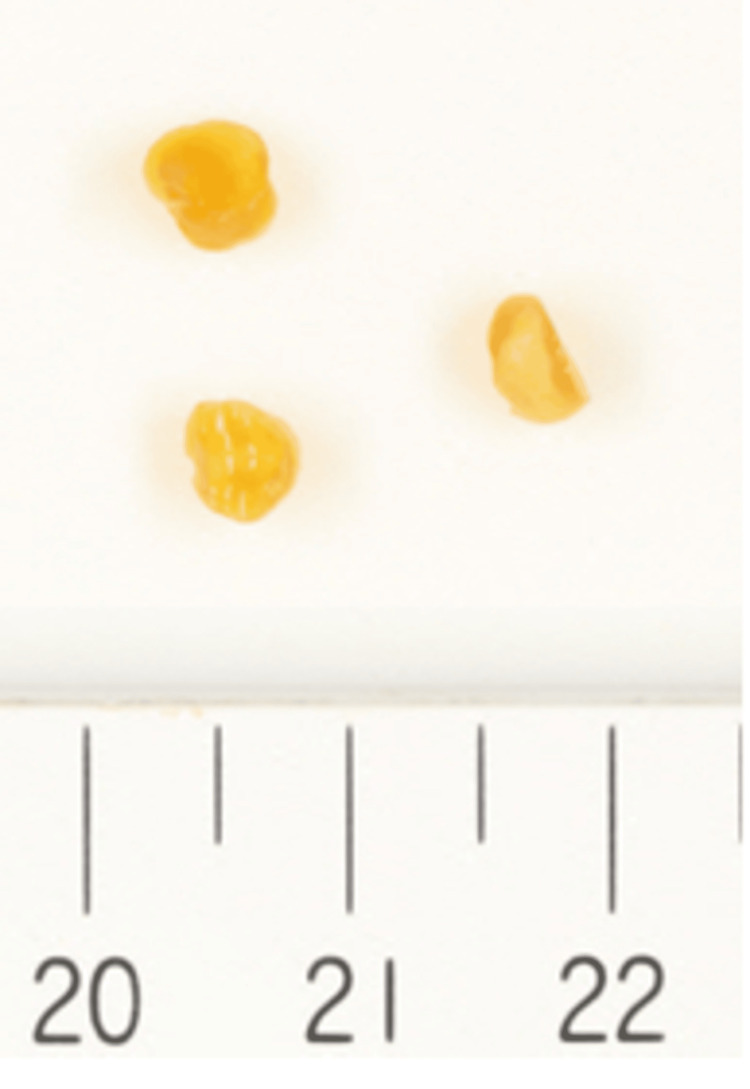
Foreign substances removed from the patient

An incision and drainage of the abscess were performed, and three foreign substances were removed (Figure 4). Each fragment measured approximately 3 mm in length and was clustered together without adhesion to the surrounding tissues. The removed material had a bone-like consistency and a yellowish color.

The patient received an IV infusion of ceftriaxone sodium (1 g/day) for three days, followed by oral amoxicillin (750 mg/day) for one week, starting on the fourth day after the initial visit. Swelling and pain subsided immediately after the removal of the foreign substances. Cone beam CT (CBCT) confirmed the absence of any residual foreign material. One month after the initial visit, the patient had achieved complete remission.

**Figure 5:**
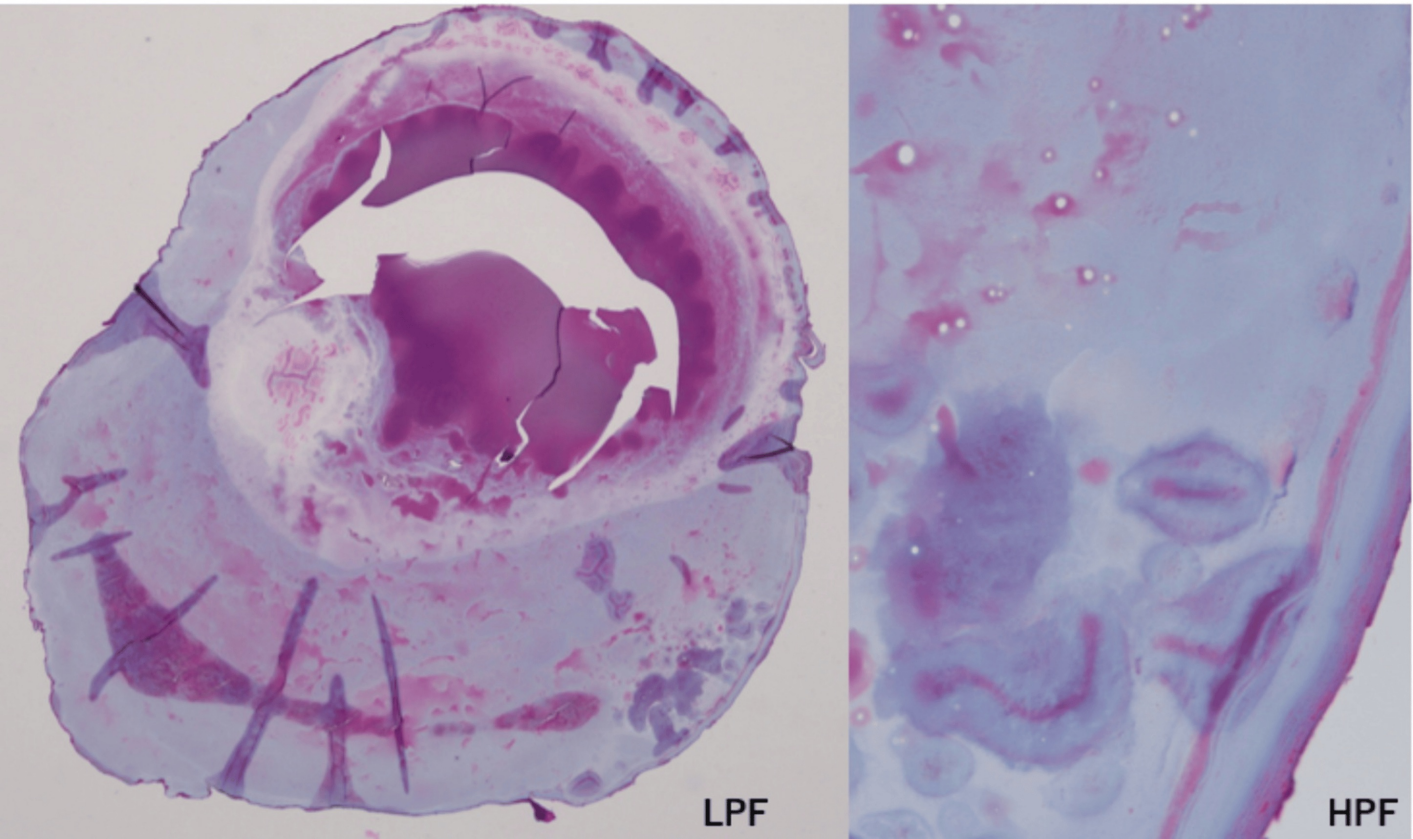
Pathological findings of the patient H&E-stained sections show that the substances lack the concentric structures typical of calculi.

Pathological examination revealed a partial concentric layer; however, typical structures such as bone, cartilage, or sialolithiasis were not present (Figure 5). Based on these findings, a definitive diagnosis of dystrophic calcification was made.

## Discussion

Dystrophic calcification is commonly triggered by injury, disease, or aging. It typically occurs in the heart, skeletal muscles, and kidneys, while lesions in the head and neck are extremely rare. To date, only three cases have been reported where dystrophic calcification was found in the masseter muscle or parotid mass (Table 2) [2,3,11].

**Table 2:** Reported cases of dystrophic calcification in the head and neck region, including the present case CBCT: cone beam CT; OPG: orthopantomography

Case	Author	Year	Age/sex	Past medical history	Affected region	Tests performed	Blood test results	Treatment	Calcified mass size
1	Walulik et al. [2]	2023	12/F	None	Left masseter muscle (two calcified masses)	USG, OPG, CBCT, and blood test	Normal	Excisional biopsy	6 mm and 8 mm
2	Chislett et al. [3]	2016	7/F	Seasonal allergic rhinitis	Left parotid gland and mandibular angle (three calcified masses)	USG, CT, and MRI	-	Excisional biopsy	5 mm, 15 mm, and 15 mm
3	Kim et al. [11]	2017	26/F	None	Left masseter muscle (three calcified masses)	USG, OPG, CT, and blood test	Normal	Excisional biopsy	4 mm, 5 mm, and 6 mm
4	Our case	2025	54/M	Disc herniation, narrowing of the disk space, and high blood pressure	Right buccal mucosa (three calcified masses)	USG, OPG, and blood test	Normal	Excisional biopsy	3 mm, 3 mm, and 3 mm

In Case 1, the patient had an interstitial lesion in the left masseter muscle and was referred from another clinic. USG, OPG, CBCT, and blood tests confirmed the presence of calcified masses, and the patient underwent an excisional biopsy. In Case 2, the patient presented with a mass anterior to the left mandibular angle. USG, CT, and MRI revealed calcified masses, leading to treatment by excisional biopsy. In Case 3, the patient reported a hardened sensation in her left cheek. USG, OPG, CT, and blood tests confirmed calcified masses, and an excisional biopsy was performed.

In these cases, the patients themselves noticed their symptoms, and diagnostic imaging, blood tests, and biopsies led to the diagnosis of dystrophic calcification. In the present case, however, the patient experienced pain and swelling, prompting abscess drainage and the removal of foreign substances. Here, dystrophic calcification occurred in the submucosal area, near the oral cavity, making it more susceptible to bacterial invasion from wounds caused by bites or trauma. To our knowledge, no other cases in the English literature have reported dystrophic calcification with concurrent infection.

In the evaluation of head and neck lesions, dystrophic calcification must be distinguished from conditions such as calcifying lymph nodes, phleboliths, sialolithiasis, and myositis ossificans [2,11,12].

Calcifying lymph nodes develop when lymph nodes in the head and neck enlarge due to inflammation, become fibrotic, and subsequently accumulate mineral deposits. As a result, they are classified as metastatic calcification [10]. This condition is often asymptomatic [13]. Radiographically, calcified lymph nodes appear as single or multiple irregular calcifications with a cauliflower-like shape and varying opacity levels [14]. Histological analysis suggests that these lymph nodes contribute to chronic inflammation by increasing lymphocyte and plasma cell counts [15]. Additionally, tuberculosis and malignancies are known to frequently induce lymph node calcification.

Phleboliths are calcified thrombi that arise from hemangiomas and developmental vascular malformations, typically forming within the soft tissues of the head and neck. Radiographically, they appear as round or oval calcified nodules, often exhibiting concentric rings resembling onion slices [11].

Another differential diagnosis is sialolithiasis, one of the most common calcification-related diseases in the head and neck region [11]. It is associated with clinical symptoms such as swelling and pain during eating. While OPG is useful for diagnosis, sialoliths can sometimes be superimposed on the teeth or mandible, making detection challenging [16]. Non-CECT typically reveals salivary stones as high-signal calcifications causing post-obstructive duct dilatation. Pathologically, these calculi exhibit concentric lamination, though their structure is often irregular due to inconsistent formation patterns [17]. Salivary stones primarily consist of calcium phosphate and carbonate, with minor amounts of magnesium, potassium, and ammonium.

Myositis ossificans is an ectopic bone formation disorder characterized by trismus. Its pathogenesis remains unclear, though it is believed to result from injury or extensive muscle strain [11]. Radiographically, it presents as linear streaks aligned with normal muscle fibers [13], while histological findings show benign, mature ossification without signs of inflammation [12].

In the present case, calcifying lymph nodes and myositis ossificans were excluded, as the patient had no history of malignancy, tuberculosis, or trauma. Sialolithiasis was also ruled out due to the swelling’s location, which was far from the major salivary glands. Furthermore, radiographic and pathological findings ruled out all other differential diagnoses.

The most effective methods for evaluating hard tissue are X-ray and CT. Although OPG can be obtained quickly, it often produces obstructive shadows. CECT is useful for assessing the extent of inflammation and abscess location. In this case, OPG did not reveal any abnormalities, so based on local findings and blood test results, CECT was considered. However, due to the patient’s severe hypertension, CECT was not performed. Instead, USG was used to successfully identify the abscess and foreign substances. While USG requires operator expertise, it is a simple, noninvasive technique that avoids radiation exposure. In this case, it proved to be highly valuable in diagnosing dystrophic calcification.

## Conclusions

Dystrophic calcification results from trauma, infection, or inflammation and occurs without a mineral imbalance. Here, we presented a case of a man with a buccal abscess and dystrophic calcification in the head and neck region, a relatively rare occurrence. In this case, a definitive diagnosis of dystrophic calcification with local infection was achieved through blood tests, USG, and pathological examination. For accurate diagnosis, a thorough medical history should be obtained, and appropriate examinations and imaging techniques should be carefully selected.
